# Assisted suicide in Austria: implications for media reporting

**DOI:** 10.1007/s00508-025-02685-6

**Published:** 2026-01-07

**Authors:** Thomas Niederkrotenthaler, Thomas Kapitany

**Affiliations:** 1 Center for Public Health, Department of Social and Preventive Medicine, Unit Public Mental Health Research, Medical University of Vienna, Vienna, Austria; 2 Crisis Intervention Center Vienna, Vienna, Austria

**Keywords:** Assisted suicide, Media reporting, Media guidelines, Suicide prevention, Austria

## Abstract

Assisted suicide is an emerging phenomenon in many western countries including Austria, with important implications for public mental health. From a suicide prevention and public health standpoint, it is essential that assisted suicide should neither be stigmatized nor romanticized or presented as the only option to address existential suffering. Balanced reporting is key to educate the public about available options at the end of life including palliative care.

The law legalizing assisted suicide (Sterbeverfügungsgesetz) has been in force in Austria since January 2022. Data from Statistics Austria show that after 54 assisted suicides in 2022 and 98 in 2023, the number rose further to 112 in 2024 [1]. Overall, assisted suicide still accounts for a relatively small number of deaths compared to unassisted suicide (in 2024 1219 people died from unassisted suicide). It is very likely, however, that the number of assisted suicides will increase in the coming years as public awareness of the law continues to grow. Data from countries where assisted suicide has been legal for longer periods corroborate that the potential is much higher than the numbers currently seen in Austria: in Belgium, for example, with a population of 11.9 million, assisted suicide has been legal since 2002. While 206 people died by assisted suicide in that country in 2003, the number was up to 3423 in 2023 [2], which is approximately 3.1% of all deaths and significantly more than the number of unassisted suicides which are comparable to the number of unassisted suicides in Austria.

Unlike unassisted suicide, where effective prevention is primarily measured in a reduction in suicide rates, the primary prevention goal for assisted suicide is more complex. With the new legal option of assisted suicide, increases in assisted suicides are also due to the sheer fact that an additional legal option for dealing with existential suffering at the end of life has been established. The public mental health problem is thus not *per se* an increase in the number of people who are interested in or use this option, but rather if existential decisions about life, dying and death are made on the basis of false, distorted, or one-sided information, in response to explicit or implicit pressure and/or based on an impression that assisted suicide is the only way to deal with suffering at the end of life. Importantly, it is widely acknowledged in psychiatric and mental health literature that temporary death wishes are frequent in the context of serious illness. This makes adequate and balanced media information even more essential, especially given that suicidal thoughts are often temporary in psychosocial crises and a potential symptom of many mental illnesses [3]. In the current media reporting, alternative options such as palliative care are often not mentioned or only mentioned briefly [4], which can indirectly create pressure to embrace assisted suicide as the best and only solution to end suffering. This contradicts the social consensus that assisted suicide should not be the first option but rather a last resort and additional option for individuals experiencing existential suffering at the end of life.

Media reports that portray suicide as the only solution, romanticize suicide or report on the suicides of prominent individuals can lead to imitation effects [5, 6]. A meta-analysis showed that after sensationalist reports about celebrity suicides, the number of suicides increased by approximately 13% in the following 4–8 weeks [7]. The same applies to novel methods of suicide. It is known that media reports about novel methods of suicide can onset the spreading of those methods [8]. A similar phenomenon was observed in Austria in the 1980s particularly in relation to suicides and suicide attempts in the Vienna subway [9, 10]. Only when the media became more cautious in their reporting on the new method of suicide, due to the introduction of media guidelines educating journalists how to report responsibly about suicide, subway suicides and suicide attempts declined by up to 80%. Although there are no specific original research studies that have analyzed the imitation effect specifically for assisted suicide, with the exception of one case study from Switzerland [11], it appears likely that the social and psychological mechanisms for imitation effects also apply in principle to assisted suicide, and that sensationalist reporting has a strong advertising effect.

It is not only the risk of imitation effects, however, that call for a sensitive approach to the topic of reporting on and portraying assisted suicide. A responsible approach is also necessary to provide balanced information on the issue, to enable different aspects to be communicated, including the often overlooked issue of dying with dignity. In particular, there is still little focus on palliative options in the current discussion [4]. Groups of people who might consider assisted suicide are very heterogeneous in terms of demographic, socioeconomic aspects, life circumstances and mental health. Many of them do not seek media spotlight themselves but rely on media sources, and sometimes also their friends and relatives in their decision process. It would be very desirable to increase the visibility of people from different walks of life who have found ways of dying with dignity beyond assisted suicide in the media discourse. The Papageno effect shows that reporting on people who have overcome suicidal thoughts reduces suicidality in others at risk of suicide. Such reports in connection with assisted suicide might also be helpful for some people who are considering suicide assistance. Portrayals that are not restricted to individuals telling their stories of why they decided for assisted suicide but feature also other types of coping with existential suffering can provide important models to think more comprehensively about options and finding a path that is right for them [12, 13].

Another relevant aspect is the timing of the reporting. In the case of the assisted suicide of Nikolaus Glattauer, which was released on *Newsflix.at* and in the weekly newspaper *Falter* in September 2025, a few days before the suicide took place, a journalistic taboo was broken [14]. The publication of this suicide announcement made highly personal considerations a topic of debate on social media and in the reader forums of the media outlets reporting on the announcement. From a prevention perspective, this approach of publishing a suicide announcement is extremely problematic. On the one hand, suicide announcements have a strong potential for imitation due to the publicity they generate, and on the other hand it is also known that many people change their minds briefly before carrying out assisted suicide. Such changes in minds are more difficult if a public media announcement of the suicide is released. In Oregon, the US state where assisted suicide has been legal under specific conditions since 1998, it is known that currently 35% of people who have already been given the lethal substance ultimately do not take it [15].

From a prevention perspective, it is important to repeatedly emphasize that the aim of suicide prevention is not to make assisted suicide a taboo subject. On the contrary, responsible media coverage is an important basis for public education. It is essential to report in a balanced manner. As highlighted in the media recommendations by the Media Working Group of the Austrian Suicide Prevention Strategy (SUPRA), which were released after the reporting on Glattauer’s suicide announcement, reporting that presents different options for individuals experiencing existential suffering is encouraged. Only balanced reporting can make a real contribution to reducing stigma and responsibly addressing the important public mental health issue of dying with dignity [16]. One-sided reporting on assisted suicide carries the risk of creating social pressure to select this form of dying.

At the same time, unbalanced reporting can create fear of the natural dying process. Worries and concerns about any anticipated suffering is likely to be reinforced. It is therefore particularly important to ensure that reports do not romanticize or even glorify suicide or devalue the natural process of dying. Simplified explanations such as “finally free from suffering” should be avoided [16]. Many examples show that assisted suicide is indeed not the only option and the only way to die with dignity, as highlighted, e.g., in the recent book by palliative care physician Eva Masel, “Gut gelaufen” (English: Worked out well) [17] or in the recent media coverage of the natural death of actor Robert Reinagl following his battle with cancer [18].

Whenever assisted suicide is discussed in the media, media recommendations for reporting on suicide should be considered and the focus should not be solely placed on individuals who have chosen assisted suicide. Austria is, to the best of our knowledge, the first country worldwide which has included guidance on the reporting of assisted suicide in the national media guidelines for the reporting on suicide. Other countries might add a similar section on assisted suicide in their guidelines in order to educate media professionals about the need of responsible reporting of assisted suicide. Psychosocial support services and counselling services for people in crisis and their relatives should always be mentioned. For a list of current help services in Austria see www.suizid-praevention.gv.at. The excellent options offered by palliative medicine should also be highlighted. Media professionals who observe these principles make a significant contribution to suicide prevention and to the issues of coping with suffering as well as dying and death with dignity.
